# An analysis of death trends in Argentina, 1990-2017, with emphasis on the effects of economic crises

**DOI:** 10.7189/jogh-10-020441

**Published:** 2020-12

**Authors:** Alejandro Macchia, Javier Mariani, Daniel Nul, Hugo Grancelli, Gianni Tognoni, Hernán C Doval

**Affiliations:** 1 GESICA Foundation, Buenos Aires, Argentina; 2 Fondazione IRCCS Ca' Granda, Ospedale Maggiore Policlinico, Milano

## Abstract

**Background:**

Economic crises have heterogeneous effects on population-level mortality between high- and low- or middle-income countries. Argentina – a middle income country- has suffered economic crises repeatedly over the past 30 years and is a model case study for the effects of economic crises on mortality.

**Methods:**

Over 28 years (1990-2017), all death records in Argentina were analysed at the most disaggregated level possible (departments, that is, second-level administrative divisions). Age-and-sex-standardized all-cause mortality, premature death (<75 years) and the probability of death at different ages for both the entire population and each socio-economic quintile were calculated by level of unsatisfied basic needs (UBNs). Standardized rates are reported as biannual average and 95% confidence interval.

**Results:**

Considered globally since the beginning of the series and using the 1990-1 biennium as a reference category, the standardized death rate was significantly reduced from biennium 2 (1992-3) to biennium 14 (2016-7), interrupted by two statistically significant increases in mortality, in years 2002-3 and 2016-7. In 2002-3, women had greater increase in mortality than men, and in 2016-7, even more so. The probability of dying before 75 years of age increased significantly in the last biennium, mostly among people between 50 and 74 years in the most deprived quintiles.

**Conclusions:**

Despite significant overall improvement over time, economic crises impose severe increases in mortality, especially among vulnerable groups such as the poor, the elderly, and women.

Although rates of general and premature death are tending to decrease in most of the world [1,2], including middle and low-income countries [3], this characterization describes average behaviour, and usually dismisses two complementary phenomena. In the first place, these averages mask the deep social differences in death rate that exist within each society [4]; second, recent estimates note a decline and even a reversal of these previously reported improvements, particularly in the United States and the United Kingdom [5-8]. These two phenomena are intertwined: the widening gaps between social groups and the slowdown and reversal of improvements are both complex and multifactorial, but economic policies in general and fiscal austerity in particular play a role in each [9,10]. The role of income distribution [11] and more broadly that of macroeconomic policies [12] both strongly influence mortality trends. However, the studies that monitor these trends and associations normally look at high-income countries, and only recently has a contribution to this discussion come from low- and middle-income countries (LMICs) [13], which are precisely those that are most vulnerable to the fluctuations of social and economic policies and conditions. Data that show a significant early change in the trend of mortality in an entire society are usually taken with disbelief and disapproval by political authorities [14] and are rarely published in time to influence decision-making to mitigate what is starting to happen or might eventually happen later. This report aims to show changes in the overall historical trend of mortality in Argentina over a period of 28 years (1990-2017) as well as that in the premature death rate as a result of fiscal adjustment policy and broad cuts in social policies in this period. This report focuses more on population-level epidemiology than on considering the burden of disease per se.

## METHODS

Based on data published annually by the Ministry of Health of Argentina, the number of deaths per year, age, gender and location of residence were collected at the most disaggregated level possible (departments, or second-level administrative divisions) from 1990 to 2017 (the last available). Data on the number of people resident in each department by age and sex in each year were taken from national censuses; for the intercensal periods, the calculation of the number of people was carried out using estimates and corrections from the National Institute of Statistics and Censuses. Various sensitivity analyses were also carried out that took into account different assumptions regarding population denominators for intercensal periods.

Standardized death rates by age and sex for the 2010 population were calculated using standard demographic techniques. The standardized coefficients were computed based on the proportions of different age and sex strata in the Argentine population as collected in the 2010 national census (the last available). Death data include the number of deaths by cause, gender, and age groups). The probability of death was calculated by age group and gender by year.

Socio-economic condition was characterized based on the degree of unsatisfied basic needs (UBNs) at the level of the 513 departments of Argentina using census data; each department was classified according to UBN quintile membership. The distribution and relative position of the departments among the different UBN quintiles was largely stable over the 28 years. However, for departments that did change their relative position during the intercensal period, quintile allocation was arbitrarily changed as of the fifth year (of ten) of that period. There was no department that changed by more than one quintiles during the period considered.

For analysis of the evolution of the national health budget, we used data published by the Institute of Health Metrics (IHMS). These data are publicly available on their website from 1995 onwards; therefore, the analysis of the evolution of the budget and its relation to adjusted death rates conforms to the period 1995-2017. The health budget is updated to 2018 US dollars and discriminated by financing type (public spending, private insurance, and out-of-pocket expense). Standardized death rates by sex and age are reported as bi-annual averages with corresponding 95% confidence intervals. The study complies with the ‘Guidelines for Accurate and Transparent Health Estimates Reporting’ (GATHER).

### Statistical analysis

All standardized death rates were calculated and are presented per 100 000 population for each department per biennium, with the corresponding 95% confidence intervals (95%CI).

In order to estimate the effects of time and economic crises, Poisson regression models were used. Time was the independent variable, while the standardized death rate from all causes, that of premature death, and the probability of dying at different points of life (<1 year, between 35 and 65 years, between 50 and 74 years, between 75 and 84 years) were dependent variables. To evaluate the presence of differential effects of economic crises between genders, interaction terms of time by gender were added to the models.

All analyses were performed considering periods of two years (biennia), and each of them (for each of the outcomes) was compared with the first biennium of the series (1990-1), with the last one (2016-7), and with the immediately preceding one.

To analyse the relationship of public, private, and out-of-pocket financing of health costs with each of the outcomes studied, linear regression models were fitted, with death rates as dependent variables and the components of health budget as independent variables, with each component in a separate model. All analyses were two-tailed, and a *P* value <0.05 was considered statistically significant.

All analyses were conducted using R version 3.6.1 for Windows (R Foundation for Statistical Computing, Vienna).

### Ethics

Since all analyses were conducted using publicly available anonymous data no ethical approval was necessary for present study.

## RESULTS

### Evolution of the standardized death rate: 1990-2017

**Figure 1:**
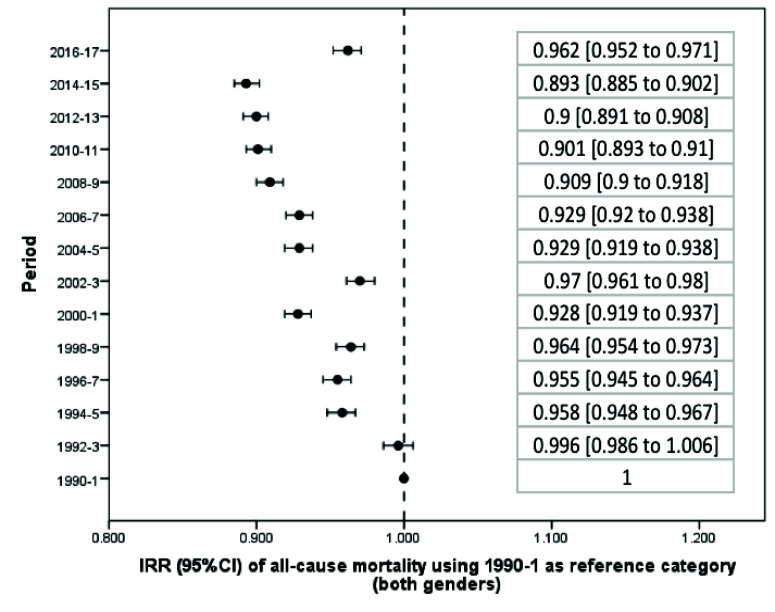
Change in the average of the standardized death rate per biennium (both genders).

**Figure 2:**
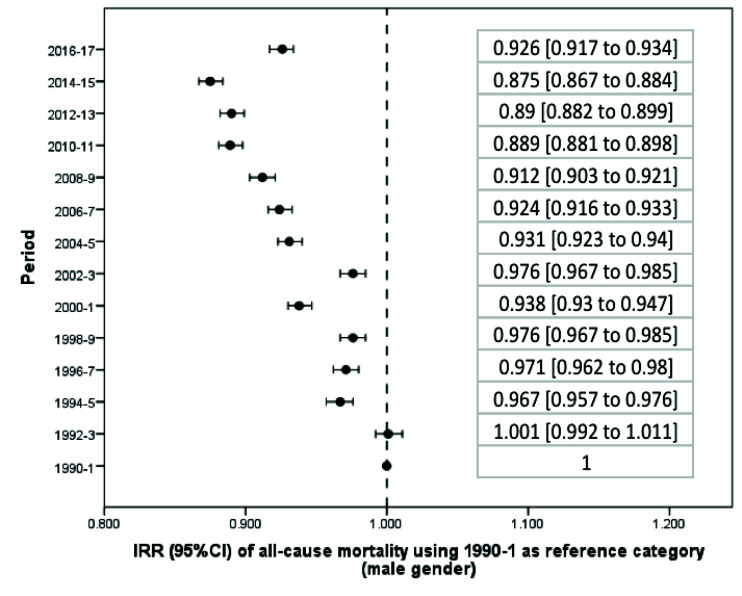
Change in the average of the standardized death rate per biennium (male gender).

**Figure 3:**
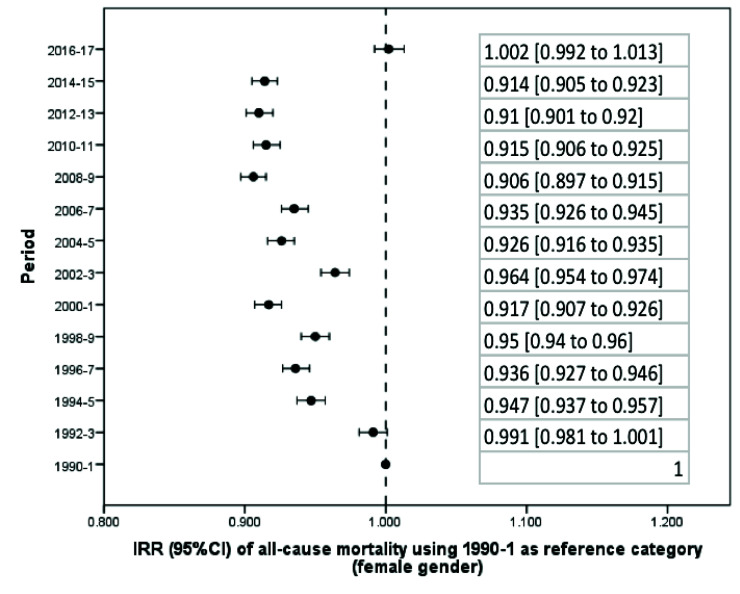
Change in the average of the standardized death rate per biennium (female gender).

**Figure 4:**
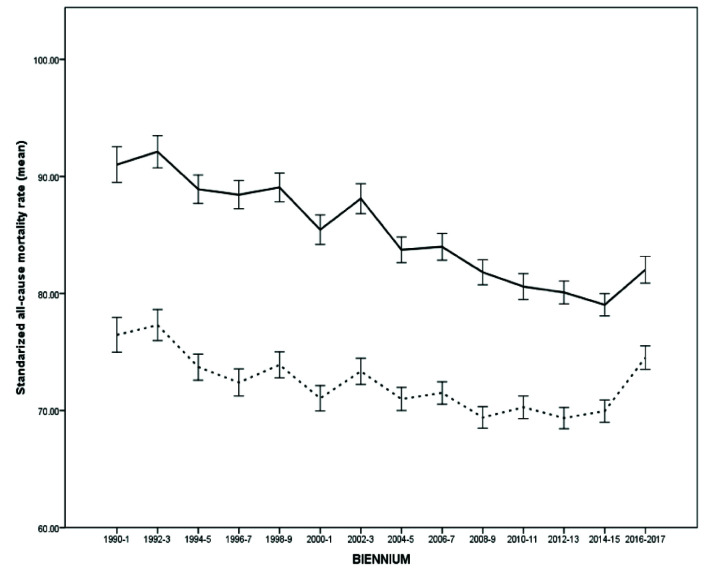
Change in the standardized average death rate in men (continuous line) and women.

From 1990 to 2017, there was a significant reduction in the average standardized death rate in Argentina (**Table 1**). Taking the first biennium as a reference category, beginning in 1992-3 overall mortality progressively decreased until the 2016-7 biennium, whose incidence rate in relation to the first biennium: incidence rate ratio, IRR, and 95% confidence interval (CI) was 0.962 (0.952 to 0.971) (Figure S1 and Table S1 in the **Online Supplementary Document**). The reduction in mortality was significant both globally (**Figure 1**) and in the male (**Figure 2**) and female (**Figure 3**) genders. However, in women there was a flattening of the decrease from 2008 onwards, a phenomenon not observed among men (**Figure 4**).

**Table 1:** Change in the standardized mortality rate (mean, 95% confidence interval, CI) by gender in Argentina from 1990 to 2017

Period	Both genders	Males	Females
1990-1	81.66 (80.34 to 82.97)	88.36 (86.85 to 89.87)	75.30 (73.78 to 76.82)
1992-3	81.35 (80.09 to 82.62)	88.46 (87.04 to 89.88)	74.61 (73.13 to 76.09)
1994-5	78.17 (77.06 to 79.29)	85.43 (84.13 to 86.74)	71.28 (69.99 to 72.58)
1996-7	77.95 (76.81 to 79.09)	85.80 (84.48 to 87.12)	70.49 (69.21 to 71.77)
1998-9	78.69 (77.60 to 79.78)	86.23 (84.91 to 87.55)	71.53 (70.33 7 to 2.74)
2000-1	75.79 (74.75 to 76.84)	82.92 (81.65 to 84.19)	69.03 (67.90 to 70.17)
2002-3	79.23 (78.16 to 80.31)	86.22 (84.98 to 87.47)	72.60 (71.32 to 73.88)
2004-5	75.83 (74.85 to 76.81)	82.28 (81.12 to 83.44)	69.70 (68.60 to 70.81)
2006-7	75.89 (74.91 to 76.87)	81.67 (80.54 to 82.80)	70.40 (69.31 to 71.50)
2008-9	74.23 (73.32 to 75.14)	80.58 (79.48 to 81.68)	68.21 (67.18 to 69.23)
2010-1	73.62 (72.66 to 74.57)	78.59 (77.47 to 79.72)	68.89 (67.84 to 69.94)
2012-3	73.45 (72.57 to 74.33)	78.64 (77.57 to 79.70)	68.53 (67.55 to 69.50)
2014-5	72.95 (71.97 to 73.93)	77.34 (76.32 to 78.36)	68.79 (67.39 to 70.19)
2016-7	78.54 (75.71 to 81.38)	81.78 (79.34 to 84.22)	75.47 (71.65 to 79.30)

Only in two biennia (corresponding to the years 2002-3 and 2016-7) was there a significant increase in mortality in relation to the preceding biennium (Table S2 in the **Online Supplementary Document**). During 2002-3 the standardized mortality rate (IRR) increased from 75.79 (95% CI = 74.75 to 76.84) to 79.23 (95% CI = 78.16 to 80.31) per 100 000 people per year, which corresponds to an increase of 1045 deaths (95% CI = 1035 to 1056). During the last available biennium, corresponding to 2016-7, the rate increased from 72.95 (95% CI = 71.97 to 73.93) to 78.54 (95% CI = 75.71 to 81.38) per 100 000 people per year, or 1077 deaths (95% CI = 1066 to 1087). Both peaks correspond to economic crises, including currency devaluation and budget cuts. In fact, using 2016-7 as a reference category, the standardized death rate in that biennium was only exceeded by those obtained in the first two biennia of the series (Table S3 in the **Online Supplementary Document**). In addition, the increase in the mortality rate in the last biennium was higher in women (IRR = 1097, 95% CI = 1086 to 1108) than in men (IRR = 1058, 95% CI = 1047 to 1068) (Table S2 in the **Online Supplementary Document**); while in the case of men, the current (2016-7) mortality rate is exceeded in 2002-3 and previous records, in women the current mortality rates are the highest in the last 28 years (Table S3 in the **Online Supplementary Document**).

### Evolution of the standardized rate of premature death

The reduction of premature death (<75 years) also showed a progressive decrease (**Table 1**), and the relative decrease compared to the beginning of the series was greater in the case of deaths before 75 years (0.816, 95% CI = 0.805 to 0.826) than in the death rate considered globally (Table S4 in the **Online Supplementary Document**). The reduction of premature death was similar in men (0.813, 95% CI = 0.804 to 0.823) and in women (0.819, 95% CI = 0.807 to 0.832). As with general death rates, the decline in the premature death rate was interrupted only in periods of economic crises, corresponding to 2002-3 (1026, 95% CI = 1012 to 1039) and 2016-7 (1057, 95% CI = 1042 to 1071) (Table S5 in the **Online Supplementary Document**). In 2002-3, the increase in the rate of premature death was similar in men and women, while in the crisis of 2016-7 the increase in premature death was significantly greater in women (1082, 95% CI = 1065 to 1.100) than in men (1041, 95% CI = 1028 to 1054) (Table S5 in the **Online Supplementary Document**). Although the rate of premature death in the last biennium was significantly higher than in the previous three biennia, the differences were smaller than what was seen with general mortality (Table S6 in the **Online Supplementary Document**).

In fact, although the probability of dying before age 75 increased significantly in the last biennium relative to the preceding biennium (1038, 95% CI = 1016 to 1061) (Table S7 in the **Online Supplementary Document**), this phenomenon was not accompanied by an increase in mortality <1 year (0.939, 95% CI = 0.814 to 1.082) (Table S8 in the **Online Supplementary Document**), which in the last biennium was the lowest in the entire series (mean and standard deviation): 0.88% ± 0.42%. Likewise, the probability of dying between 35 and 65 years declined significantly throughout the series and did not increase significantly in the last two years (1019, 95% CI = 0983 to 1055) (Table S9 in the **Online Supplementary Document**).

The increase in general mortality was due to an increase in both premature death and death in the elderly; however, the increase in premature death contributed less than the increase in death in the elderly.

### Dead by age, sex, and socioeconomic status in the last two biennia

**Figure 5:**
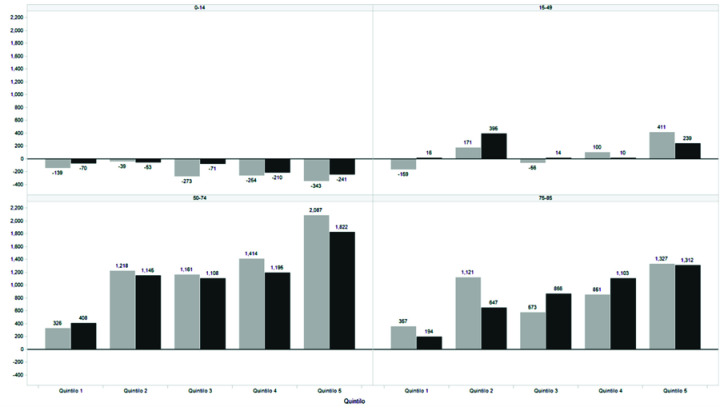
Comparison of number of deaths by age group and CSE quintile (biennium 2014-5 vs biennium 2016-7).

A total of 33 806 more deaths were recorded in the last biennium, 2016-7 (n = 690 086) than in the immediately preceding biennium (n = 656 280). In these two biennium, while the total population increased by 2.14%, mortality increased by 5.15%. Of all these deaths, 18 616 were in people <85 years. While for the group from 0 to 14 years of age the calculated population went from 21.64 million to 21.82 million people (+0.83%), mortality decreased from 19 622 to 17 856 (-9.0%). Between the ages of 15 and 49 the population increased by 2.19% (43.17 million to 44.12 million) while mortality was +0.99% (from 63 416 to 64 047). Between 50 and 74 years the population increased from 17.08 million to 17.62 million (+3.18%) while mortality increased from 229.087 to 240.653 (+5.04%). Finally, people between 75 and 84 years old increased in number from 2.87 million to 2.97 million (+3.49%) while mortality increased from 191.406 to 199.591 (+4.27%). Most of those deaths were concentrated in the 50-74 age group and belonged to the 4^th^ and 5^th^ UBN quintiles (**Figure 5**).

### Public financing of health costs and changes in mortality

The amount invested in public financing of health care showed significant relationships with general death rates (*P* = 0.002) and premature death (*P* < 0.0001), showing that the years in which the state allocated more money to assistance had lower mortality. This relationship was not significant with the provision of money through private assistance for death in general (*P* = 0.245) or for premature death (*P* = 0.717). Finally, there was a statistically significant relationship between out-of-pocket expenditure and premature mortality (*P* = 0.045) although not for total mortality (*P* = 0.217).

## DISCUSSION

The results of this historical series of mortality data for the years 1990-2017 in Argentina show a significant reduction in standardized rates of death, premature death, and infant mortality, with the last biennium being the period of lowest death rate under 1 year, after a sustained rate of decline throughout the series. All these data are in line with international metrics, reflecting the same signals of progress in general and in Latin America in particular [3,15]. But the mere publication and dissemination of average data can be distorting [16] and, in fact, the results of this analysis reveal particular aspects that we believe deserve to be discussed.

Progress in reducing standardized death rates was interrupted in the periods of economic crisis corresponding to the 2002-3 and 2016-7 biennia. These crises had a much greater impact than the secular trends, so that periods of crisis in fact cancel many years of moderate progress. In fact, the mortality rates of women in the last two years in Argentina are similar to those almost 30 years ago.

The standard approaches to addressing economic crisis include measures ranging from adjustment policies to those aimed at boosting production. These measures do not have an invariable result in health terms, and in fact some authors have found that austerity increases mortality [17-23] while others have found an opposite result [24-30]. The results seem then dependent on the characteristics and strength of the systems to which these measures are applied [12,31].

In the case of Argentina—where the public health system is chronically deficient in terms of both economic and demand response—the results show that austerity models produce even more serious deleterious consequences than in high-income countries. This contribution is the first to document the effects of austerity policies in Argentina.

The mechanisms by which economic crises increase mortality were not formally explored in this work. What is clear is that crises generate a reduction in public spending on health, a phenomena that in the literature has been particularly relevant when they emerge in the context of agreements with multinational credit organizations [17], leading to the imposition of austerity, which was the Argentine case in the two periods indicated. This reduction in health spending also entails a reduction in the provision of services and the purchase of materials, as well as an increase in the price of supplies and a decreased in the availability of professionals [32]. In fact, the present analysis also shows a significant relationship between public spending on health and mortality, especially affecting premature mortality, as well as an increase in early mortality associated with increase in out-of-pocket expenses, in line with what has been published [33] elsewhere.

The distribution of excess deaths during economic crises was socially conditioned. The cost of reduced health care service in lost lives was mainly concentrated in the group of people between 50 and 74 years old and the lowest socioeconomic quintiles. The probability of dying at an early age is socioeconomically conditioned in other countries as well [34]; however, there is little reliable evidence that the cost in human lives of fiscal adjustment is directly related to the vulnerability of the population.

A fact that deserves comment is that in Argentina the economic crises hit with particular impact social groups that are usually outside the accountability measurements of classical epidemiology, as the most punished people in the recent crisis were not children but middle-aged and elderly people. This was also a finding in countries such as England [8], and underlines the need to continue insisting on control policies and availability of services for groups that are not normally on the agenda of accountability programmes. Researchers of the epidemiology of preventable deaths in low- and middle-income countries should continue to monitor classic risk groups such as children and pregnant women; however, discontinuity of treatment in chronic non-communicable diseases can have devastating effects on health in other population segments too.

This work can describe what happened in the years of crisis only in Argentina and in terms of vital statistics. The work cannot claim any causality but can only describe associations. However, the most recent crisis is only the beginning of Argentina’s recent economic changes. In 2017, the economy expanded by nearly 3%, and Argentina sold $100 billion in government bonds during 2016-7, exploiting its newfound favour with the international financial set. This cash allowed the government to maintain some social programmes, although in general it pursued an agenda of reducing and balancing public spending, which represented almost 8% of GDP at the beginning of the Macri administration (2015-9). Nevertheless, since the beginning of 2018, the economy has been contracting, inflation has run above 50%, and joblessness has been stuck above 9%. Poverty afflicts a third of the population, and the figures are climbing. How this will impact mortality should be investigated in the coming years. In light of what the present almost 30-year analysis suggests, the price of what will come is likely to be disproportionately paid by the segments of society with the greatest social and economic vulnerability.

Our analysis has both strengths and limitations. Regarding the former, we believe the results are robust and clearly show the relationship between social vulnerability, economic crisis, and excess deaths in a vulnerable population. Our data are real (not projections) and complete and have a sufficient degree of granular disaggregation and temporal length. In addition, the analysis centres people and not diseases as the protagonists of economic crises. In this way, this is not an analysis of the burden of disease but rather of social vulnerability to crisis.

On the other hand, the analysis contains limitations that must be recognized. We use biannual periods, since, although we consider all deaths in Argentina over a period of 28 years, the use of annual metrics is sometimes unstable. Also, Argentina has a relatively low population and the use of a single standardized annual rate can exhibit fluctuations.

The analysis did not focus on any particular type of death, and a separate analysis was not made to differentiate deaths that might be considered avoidable from those that might not be. Although not all premature deaths are preventable, we believe that the revealed indicators are quite indicative of the relationship between social vulnerability to economic crisis and mortality.

## Additional material

Online Supplementary Document
