# Supplementary material for: An analysis of death trends in Argentina, 1990-2017, with emphasis on the effects of economic crises
Source: J Glob Health. 2020 Dec 6;10(2):020441. doi: 10.7189/jogh-10-020441 (PMC7719272; doi:10.7189/jogh-10-020441)
Supplement: Online Supplementary Document [file jogh-10-020441-s001.pdf]

An analysis of death trends in Argentina, 1990–2017, with emphasis on the effects of economic crises

Online Supplementary Documents

Table S1. Incidence rate ratio (IRR) of overall mortality as compared with first biennium.

Table S2. Incidence rate ratio (IRR) of overall mortality as compared with previous biennium.

Table S3. Incidence rate ratio (IRR) of overall mortality as compared with last biennium.

Table S4. Incidence rate ratio (IRR) of premature mortality as compared with first biennium.

Table S5. Incidence rate ratio (IRR) of premature mortality as compared with previous biennium.

Table S6. Incidence rate ratio (IRR) of premature mortality as compared with last biennium.

Table S7. Probability of dying before 75 years. Mean (95%CI) and comparison with first, last and previous biennium.

Table S8. Probability of dying before 1 year. Mean (95%CI) and comparison with first, last and previous biennium.

Table S9. Probability of dying between 35 to 65 years. Mean (95%CI) and comparison with first, last and previous biennium.

Table S1: Incidence rate ratio (IRR) of overall mortality as compared with first biennium.

		Both genders (compared with first biennium)			Males (compared with first biennium)			Females (compared with first biennium)		
Period	Biennium	IRR	95%CI	*P*	IRR	95%CI	*P*	IRR	95%CI	*P*
1990-1	1	1	.		1	.	.	1		.
1992-3	2	0.996	0.986-1.006	0.44	1.001	0.992-1.011	0.835	0.991	0.981-1.001	0.084
1994-5	3	0.958	0.948-0.967	<0.0001	0.967	0.957-0.976	<0.0001	0.947	0.937-0.957	<0.0001
1996-7	4	0.955	0.945-0.964	<0.0001	0.971	0.962-0.98	<0.0001	0.936	0.927-0.946	<0.0001
1998-9	5	0.964	0.954-0.973	<0.0001	0.976	0.967-0.985	<0.0001	0.95	0.94-0.96	<0.0001
2000-1	6	0.928	0.919-0.937	<0.0001	0.938	0.93-0.947	<0.0001	0.917	0.907-0.926	<0.0001
2002-3	7	0.97	0.961-0.98	<0.0001	0.976	0.967-0.985	<0.0001	0.964	0.954-0.974	<0.0001
2004-5	8	0.929	0.919-0.938	<0.0001	0.931	0.923-0.94	<0.0001	0.926	0.916-0.935	<0.0001
2006-7	9	0.929	0.92-0.938	<0.0001	0.924	0.916-0.933	<0.0001	0.935	0.926-0.945	<0.0001
2008-9	10	0.909	0.9-0.918	<0.0001	0.912	0.903-0.921	<0.0001	0.906	0.897-0.915	<0.0001
2010-11	11	0.901	0.893-0.91	<0.0001	0.889	0.881-0.898	<0.0001	0.915	0.906-0.925	<0.0001
2012-13	12	0.9	0.891-0.908	<0.0001	0.89	0.882-0.899	<0.0001	0.91	0.901-0.92	<0.0001
2014-15	13	0.893	0.885-0.902	<0.0001	0.875	0.867-0.884	<0.0001	0.914	0.905-0.923	<0.0001
2016-2017	14	0.962	0.952-0.971	<0.0001	0.926	0.917-0.934	<0.0001	1.002	0.992-1.013	0.638

Table S2: Incidence rate ratio (IRR) of overall mortality as compared with previous biennium.

		Both genders (compared with first biennium)			Males (compared with first biennium)			Females (compared with first biennium)		
Period	Biennium	IRR	95%CI	*P*	IRR	95%CI	*P*	IRR	95%CI	*P*
1990-1	1	1			1			1		
1992-3	2	0.996	0.986-1.006	0.44	1.001	0.992-1.011	0.835	0.991	0.981-1.001	0.084
1994-5	3	0.961	0.952-0.971	<0.0001	0.966	0.957-0.975	<0.0001	0.956	0.946-0.966	<0.0001
1996-7	4	0.997	0.987-1.007	0.561	1.004	0.995-1.014	0.366	0.989	0.978-0.999	0.033
1998-9	5	1.009	0.999-1.019	0.067	1.005	0.996-1.015	0.287	1.015	1.004-1.025	0.005
2000-1	6	0.963	0.954-0.973	<0.0001	0.962	0.953-0.971	<0.0001	0.965	0.955-0.975	<0.0001
2002-3	7	1.045	1.035-1.056	<0.0001	1.04	1.03-1.05	<0.0001	1.052	1.041-1.062	<0.0001
2004-5	8	0.957	0.948-0.966	<0.0001	0.955	0.946-0.963	<0.0001	0.96	0.951-0.97	<0.0001
2006-7	9	1.001	0.991-1.011	0.867	0.992	0.983-1.002	0.115	1.01	1-1.021	0.051
2008-9	10	0.978	0.969-0.988	<0.0001	0.987	0.977-0.996	0.006	0.968	0.959-0.978	<0.0001
2010-11	11	0.992	0.982-1.002	0.099	0.975	0.966-0.985	<0.0001	1.01	1-1.021	0.055
2012-13	12	0.998	0.988-1.008	0.667	1.001	0.991-1.011	0.874	0.995	0.984-1.005	0.302
2014-15	13	0.993	0.983-1.003	0.179	0.983	0.974-0.993	0.001	1.004	0.994-1.015	0.433
2016-2017	14	1.077	1.066-1.087	<0.0001	1.058	1.047-1.068	<0.0001	1.097	1.086-1.108	<0.0001

Table S3: Incidence rate ratio (IRR) of overall mortality as compared with last biennium.

		Both genders (compared with first biennium)			Males (compared with first biennium)			Females (compared with first biennium)		
Period	Biennium	IRR	95%CI	*P*	IRR	95%CI	*P*	IRR	95%CI	*P*
1990-1	1	1.04	1.03-1.05	<0.0001	1.08	1.07-1.091	<0.0001	0.998	0.988-1.008	0.638
1992-3	2	1.036	1.026-1.046	<0.0001	1.081	1.071-1.092	<0.0001	0.989	0.979-0.999	0.026
1994-5	3	0.996	0.986-1.006	0.387	1.044	1.034-1.054	<0.0001	0.945	0.935-0.954	<0.0001
1996-7	4	0.993	0.983-1.002	0.143	1.049	1.039-1.059	<0.0001	0.934	0.925-0.943	<0.0001
1998-9	5	1.002	0.992-1.012	0.701	1.054	1.044-1.064	<0.0001	0.948	0.938-0.957	<0.0001
2000-1	6	0.965	0.956-0.975	<0.0001	1.014	1.004-1.023	0.004	0.915	0.905-0.924	<0.0001
2002-3	7	1.009	0.999-1.019	0.071	1.054	1.044-1.064	<0.0001	0.962	0.952-0.971	<0.0001
2004-5	8	0.965	0.956-0.975	<0.0001	1.006	0.997-1.016	0.201	0.924	0.914-0.933	<0.0001
2006-7	9	0.966	0.957-0.976	<0.0001	0.999	0.989-1.008	0.764	0.933	0.924-0.943	<0.0001
2008-9	10	0.945	0.936-0.954	<0.0001	0.985	0.976-0.995	0.002	0.904	0.895-0.913	<0.0001
2010-11	11	0.937	0.928-0.947	<0.0001	0.961	0.952-0.97	<0.0001	0.913	0.904-0.922	<0.0001
2012-13	12	0.935	0.926-0.944	<0.0001	0.962	0.953-0.971	<0.0001	0.908	0.899-0.917	<0.0001
2014-15	13	0.929	0.92-0.938	<0.0001	0.946	0.937-0.955	<0.0001	0.912	0.903-0.921	<0.0001
2016-2017	14	1	.	.	1	.	.	1	.	.

Table S4: Incidence rate ratio (IRR) of premature mortality as compared with first biennium.

		Both genders (compared with first biennium)			Males (compared with first biennium)			Females (compared with first biennium)		
Period	Biennium	IRR	95%CI	*P*	IRR	95%CI	*P*	IRR	95%CI	*P*
1990-1	1	1	.		1	.		1	.	
1992-3	2	0.985	0.973-0.998	0.023	0.993	0.982-1.005	0.264	0.972	0.958-0.987	<0.0001
1994-5	3	0.953	0.94-0.965	<0.0001	0.962	0.951-0.973	<0.0001	0.94	0.926-0.954	<0.0001
1996-7	4	0.932	0.92-0.944	<0.0001	0.953	0.942-0.964	<0.0001	0.899	0.886-0.913	<0.0001
1998-9	5	0.92	0.908-0.932	<0.0001	0.941	0.93-0.952	<0.0001	0.886	0.873-0.9	<0.0001
2000-1	6	0.875	0.864-0.886	<0.0001	0.891	0.881-0.901	<0.0001	0.85	0.838-0.863	<0.0001
2002-3	7	0.897	0.886-0.909	<0.0001	0.913	0.903-0.924	<0.0001	0.873	0.86-0.886	<0.0001
2004-5	8	0.84	0.829-0.851	<0.0001	0.862	0.851-0.872	<0.0001	0.807	0.795-0.819	<0.0001
2006-7	9	0.837	0.826-0.848	<0.0001	0.852	0.842-0.862	<0.0001	0.812	0.8-0.825	<0.0001
2008-9	10	0.829	0.818-0.84	<0.0001	0.842	0.832-0.852	<0.0001	0.808	0.796-0.821	<0.0001
2010-11	11	0.796	0.785-0.807	<0.0001	0.805	0.796-0.815	<0.0001	0.781	0.77-0.794	<0.0001
2012-13	12	0.801	0.79-0.811	<0.0001	0.812	0.802-0.822	<0.0001	0.784	0.772-0.796	<0.0001
2014-15	13	0.772	0.761-0.782	<0.0001	0.781	0.772-0.791	<0.0001	0.757	0.745-0.769	<0.0001
2016-2017	14	0.816	0.805-0.826	<0.0001	0.813	0.804-0.823	<0.0001	0.819	0.807-0.832	<0.0001

Table S5: Incidence rate ratio (IRR) of premature mortality as compared with previous biennium.

		Both genders (compared with first biennium)			Males (compared with first biennium)			Females (compared with first biennium)		
Period	Biennium	IRR	95%CI	*P*	IRR	95%CI	*P*	IRR	95%CI	*P*
1990-1	1	1.00			1	.		1	.	
1992-3	2	0.985	0.973-0.998	0.023	0.993	0.982-1.005	0.264	0.972	0.958-0.987	<0.0001
1994-5	3	0.967	0.954-0.98	<0.0001	0.968	0.957-0.98	<0.0001	0.967	0.952-0.981	<0.0001
1996-7	4	0.978	0.966-0.991	<0.0001	0.991	0.98-1.003	0.138	0.957	0.943-0.972	<0.0001
1998-9	5	0.987	0.974-1	0.046	0.987	0.976-0.999	0.027	0.986	0.971-1.001	0.062
2000-1	6	0.951	0.939-0.964	<0.0001	0.947	0.936-0.958	<0.0001	0.959	0.945-0.974	<0.0001
2002-3	7	1.026	1.012-1.039	<0.0001	1.025	1.013-1.037	<0.0001	1.026	1.011-1.042	0.001
2004-5	8	0.936	0.924-0.948	<0.0001	0.943	0.932-0.954	<0.0001	0.924	0.91-0.938	<0.0001
2006-7	9	0.996	0.983-1.01	0.567	0.989	0.977-1.001	0.073	1.007	0.991-1.023	0.383
2008-9	10	0.991	0.978-1.004	0.19	0.988	0.977-1.001	0.06	0.995	0.98-1.011	0.548
2010-11	11	0.96	0.947-0.973	<0.0001	0.956	0.944-0.968	<0.0001	0.967	0.951-0.982	<0.0001
2012-13	12	1.006	0.992-1.02	0.404	1.008	0.995-1.02	0.213	1.003	0.987-1.019	0.698
2014-15	13	0.964	0.951-0.978	<0.0001	0.962	0.95-0.974	<0.0001	0.966	0.95-0.981	<0.0001
2016-2017	14	1.057	1.042-1.071	<0.0001	1.041	1.028-1.054	<0.0001	1.082	1.065-1.1	<0.000

Table S6: Incidence rate ratio (IRR) of premature mortality as compared with last biennium.

		Both genders (compared with first biennium)			Males (compared with first biennium)			Females (compared with first biennium)		
Period	Biennium	IRR	95%CI	*P*	IRR	95%CI	*P*	IRR	95%CI	*P*
1990-1	1	1.226	1.21-1.243	<0.0001	1.23	1.215-1.244	<0.0001	1.22	1.202-1.239	<0.0001
1992-3	2	1.208	1.192-1.224	<0.0001	1.222	1.207-1.236	<0.0001	1.187	1.169-1.205	<0.0001
1994-5	3	1.168	1.153-1.184	<0.0001	1.183	1.169-1.197	<0.0001	1.147	1.129-1.165	<0.0001
1996-7	4	1.143	1.128-1.158	<0.0001	1.172	1.158-1.186	<0.0001	1.098	1.081-1.115	<0.0001
1998-9	5	1.128	1.113-1.143	<0.0001	1.157	1.143-1.171	<0.0001	1.082	1.065-1.099	<0.0001
2000-1	6	1.073	1.058-1.087	<0.0001	1.096	1.083-1.109	<0.0001	1.038	1.022-1.054	<0.0001
2002-3	7	1.101	1.086-1.115	<0.0001	1.123	1.11-1.137	<0.0001	1.065	1.049-1.082	<0.0001
2004-5	8	1.03	1.016-1.044	<0.0001	1.059	1.047-1.072	<0.0001	0.984	0.969-1	0.049
2006-7	9	1.026	1.012-1.04	<0.0001	1.048	1.035-1.061	<0.0001	0.991	0.976-1.007	0.275
2008-9	10	1.017	1.003-1.031	0.018	1.036	1.023-1.048	<0.0001	0.987	0.971-1.002	0.091
2010-11	11	0.976	0.963-0.99	0.001	0.99	0.978-1.003	0.122	0.954	0.939-0.969	<0.0001
2012-13	12	0.982	0.968-0.995	0.009	0.998	0.986-1.011	0.769	0.957	0.942-0.972	<0.0001
2014-15	13	0.946	0.933-0.96	<0.0001	0.961	0.949-0.973	<0.0001	0.924	0.909-0.939	<0.0001
2016-2017	14	1	.		1	.		1	.	

Table S7: Probability of dying before 75 years. Mean (95%CI) and comparison with first, last and previous biennium.

		Probability of dying before 75		IRR (95%CI) against first biennium		IRR (95%CI) against last biennium		IRR (95%CI) against precedent biennium	
Period	Biennium	Mean	95% CI	IRR	95% CI	IRR	95% CI	IRR	95% CI
1990-1	1	51.69	50.65-50.65	1	.	1.184	1.159-1.209	1	.
1992-3	2	51.55	50.56-50.56	0.997	0.977-1.018	1.18	1.156-1.206	0.997	0.977-1.018
1994-5	3	50.53	49.66-49.66	0.977	0.957-0.997	1.156	1.132-1.181	0.98	0.959-1
1996-7	4	49.74	48.83-48.83	0.962	0.942-0.982	1.138	1.115-1.163	0.985	0.964-1.005
1998-9	5	49.06	48.15-48.15	0.949	0.93-0.969	1.124	1.1-1.148	0.987	0.967-1.008
2000-1	6	46.90	46.01-46.01	0.907	0.888-0.926	1.074	1.051-1.097	0.956	0.936-0.976
2002-3	7	47.51	46.63-46.63	0.919	0.9-0.939	1.088	1.066-1.111	1.014	0.993-1.035
2004-5	8	45.27	44.51-44.51	0.876	0.858-0.894	1.037	1.015-1.059	0.953	0.933-0.973
2006-7	9	44.52	43.73-43.73	0.861	0.843-0.879	1.019	0.997-1.041	0.983	0.962-1.004
2008-9	10	43.77	43.01-43.01	0.847	0.829-0.865	1.002	0.981-1.024	0.984	0.963-1.005
2010-11	11	42.86	42.08-42.08	0.829	0.811-0.846	0.981	0.96-1.002	0.979	0.958-1
2012-13	12	42.67	41.9-41.9	0.825	0.808-0.843	0.977	0.956-0.998	0.996	0.975-1.018
2014-15	13	42.07	41.33-41.33	0.814	0.797-0.831	0.963	0.943-0.984	0.986	0.965-1.008
2016-2017	14	43.70	42.8-42.8	0.845	0.827-0.863	1	.	1.038	1.016-1.061

Table S8: Probability of dying before 1 year. Mean (95%CI) and comparison with first, last and previous biennium.

		Probability of dying before 75		IRR (95%CI) against first biennium		IRR (95%CI) against last biennium		IRR (95%CI) against precedent biennium	
Period	Biennium	Mean	95% CI	IRR	95% CI	IRR	95% CI	IRR	95% CI
1990-1	1	2.383	2.29-2.477	1	.	2.486	2.198-2.812	1	.
1992-3	2	2.157	2.065-2.249	0.896	0.811-0.989	2.227	1.965-2.524	0.896	0.811-0.989
1994-5	3	2.002	1.92-2.083	0.835	0.754-0.924	2.075	1.829-2.356	0.932	0.84-1.034
1996-7	4	1.836	1.751-1.922	0.774	0.699-0.858	1.925	1.694-2.188	0.928	0.833-1.033
1998-9	5	1.749	1.667-1.831	0.734	0.661-0.814	1.824	1.604-2.074	0.948	0.849-1.057
2000-1	6	1.581	1.511-1.651	0.657	0.591-0.73	1.633	1.434-1.86	0.895	0.8-1.001
2002-3	7	1.607	1.534-1.68	0.674	0.607-0.748	1.675	1.472-1.906	1.026	0.916-1.149
2004-5	8	1.432	1.371-1.493	0.583	0.523-0.65	1.449	1.269-1.655	0.865	0.77-0.972
2006-7	9	1.287	1.235-1.34	0.53	0.474-0.593	1.318	1.151-1.509	0.909	0.804-1.028
2008-9	10	1.297	1.248-1.346	0.538	0.481-0.601	1.337	1.168-1.53	1.014	0.895-1.15
2010-11	11	1.216	1.168-1.264	0.506	0.452-0.567	1.258	1.097-1.443	0.941	0.829-1.068
2012-13	12	1.061	1.019-1.102	0.434	0.385-0.489	1.079	0.936-1.243	0.857	0.75-0.98
2014-15	13	1.004	0.966-1.041	0.428	0.38-0.483	1.065	0.924-1.228	0.987	0.859-1.135
2016-2017	14	0.883	0.84-0.925	0.402	0.356-0.455	1	.	0.939	0.814-1.082

Table S9: Probability of dying between 35 to 65 years. Mean (95%CI) and comparison with first, last and previous biennium.

		Probability of dying before 75		IRR (95%CI) against first biennium		IRR (95%CI) against last biennium		IRR (95%CI) against precedent biennium	
Period	Biennium	Mean	95% CI	IRR	95% CI	IRR	95% CI	IRR	95% CI
1990-1	1	20.97	20.47-21.46	1	.	1.281	1.238-1.325	1	.
1992-3	2	21.06	20.59-21.54	1.007	0.975-1.041	1.29	1.247-1.335	1.007	0.975-1.041
1994-5	3	20.06	19.67-20.46	0.957	0.925-0.989	1.225	1.184-1.268	0.95	0.919-0.981
1996-7	4	19.79	19.38-20.21	0.945	0.914-0.977	1.21	1.17-1.252	0.988	0.955-1.021
1998-9	5	19.34	18.95-19.73	0.924	0.894-0.955	1.184	1.144-1.225	0.978	0.946-1.011
2000-1	6	18.49	18.09-18.9	0.883	0.854-0.912	1.13	1.093-1.17	0.955	0.924-0.987
2002-3	7	18.77	18.38-19.16	0.896	0.868-0.926	1.148	1.11-1.188	1.016	0.983-1.05
2004-5	8	17.81	17.45-18.16	0.851	0.823-0.88	1.09	1.054-1.128	0.95	0.918-0.982
2006-7	9	17.79	17.42-18.16	0.849	0.821-0.877	1.087	1.051-1.125	0.997	0.964-1.031
2008-9	10	17.38	17.03-17.74	0.831	0.803-0.859	1.064	1.028-1.101	0.979	0.946-1.012
2010-11	11	16.69	16.32-17.06	0.797	0.77-0.824	1.021	0.986-1.057	0.959	0.927-0.993
2012-13	12	16.65	16.27-17.03	0.796	0.769-0.823	1.019	0.984-1.055	0.999	0.965-1.034
2014-15	13	16.09	15.75-16.42	0.766	0.741-0.793	0.982	0.948-1.017	0.963	0.93-0.998
2016-2017	14	16.36	15.95-16.78	0.781	0.755-0.808	1	.	1.019	0.983-1.055
